# The association of preoperative hematologic parameters with short‐term clinical outcomes in rectal cancer: A feature importance analysis

**DOI:** 10.1002/cam4.7225

**Published:** 2024-05-22

**Authors:** Ala Orafaie, Fatemeh Shahabi, Ali Mehri, Majid Ansari, Sajjad Kasraeifar, Mahdie Ghiyasi, Maryam Saberi‐Karimian, Abbas Abdollahi, Seyyed Mohammad Tabatabaei

**Affiliations:** ^1^ Endoscopic and Minimally Invasive Surgery Research Center Mashhad University of Medical Sciences Mashhad Iran; ^2^ Department of Medical Informatics, School of Medicine Mashhad University of Medical Sciences Mashhad Iran; ^3^ Clinical Research Unit, Imam Reza Hospital, School of Medicine Mashhad University of Medical Sciences Mashhad Iran; ^4^ Lung Diseases Research Center Mashhad University of Medical Sciences Mashhad Iran

**Keywords:** clinical outcomes, hematologic parameters, machine learning, prognostic factor, rectal cancer

## Abstract

**Background:**

Various hematologic parameters have been proposed as prognostic factors in rectal cancer management, but data are conflicting and unclear. This study is designed to investigate the prognostic factor capability of preoperative hematologic parameters with postoperative morbidities and mortality in rectal cancer patients undergoing curative resection.

**Methods:**

All 200 consecutive rectal cancer patients diagnosed at Ghaem University Hospital from 2017 to 2022 were retrospectively evaluated. The receiver operating characteristic (ROC) curves and machine learning (ML) algorithms of Random Forest, Recursive Feature Elimination, simulated annealing, Support Vector Machine, Decision Tree, and eXtreme Gradient Boosting were administered to investigate the role of preoperative hematologic parameters accompanied by baseline characteristics on three clinical outcomes including surgical infectious complications, recurrence, and death.

**Results:**

The frequency of infectious complications was correlated with the surgical procedure, while tumor recurrence was significantly influenced by T stage and N stage. In terms of mortality, alongside T and N stage, the status of resection margin involvement was significantly correlated. Based on the ROC analysis, the NLR >2.69, MPV ≤9 fL, and PDW ≤10.5 fL were more classified patients to mortality status. Likewise, the PLT >220 10^9^/L, MPV ≤9 fL, PDW ≤10.4 fL, and PLR >13.6 were correlated with recurrence. However, all factors examined in this study were not significant classifiers for the outcome of surgical infectious complications. The results of ML algorithms were also in line with ROC analysis.

**Conclusion:**

According to the results of both ROC analysis and ML models, preoperative hematologic parameters are considerable prognostic factors of postoperative outcomes in rectal cancer patients, and are recommended to be monitored by clinicians to prevent unfavorable outcomes.

## 1 INTRODUCTION

Colorectal cancer (CRC) is one of the most common cancers worldwide. Patients with CRC have the fourth highest morbidity and second highest mortality rates, with more than 1 million new cases and 600,000 deaths annually.^1^, ^2^ Approximately 40% of CRC cases are diagnosed with rectal cancer.^3^ Therefore, it is necessary to identify all factors that are associated with rectal cancer morbidity and mortality.

Numerous methods have been developed to evaluate prognosis and accurately improve rectal cancer patients' survival.^4^ In recent years, various types of biochemical markers and indicators related to this malignancy's inflammatory processes and biological characteristics have surged as diagnostic and prognostic tools.^5^, ^6^, ^7^ A wide variety of preoperative hematologic parameters with different changes were evaluated and discussed in the study of CRC, such as creatinine, PLT (platelet), NLR (neutrophil‐to‐lymphocyte ratio), PLR (platelet‐to‐lymphocyte ratio), MPV (mean platelet volume), LWR (lymphocyte‐to‐white blood cell ratio), RDW (red cell distribution width), RDW‐CV (red cell distribution width‐coefficient of variation), and PDW (platelet distribution width).^4^, ^8^, ^9^, ^10^, ^11^, ^12^ However, the exploration of hematologic biomarkers associated with survival, treatment response, and prognosis of rectal cancer is still needed. This would help identify high‐risk patients requiring follow‐up treatment and could lead to an early awareness of clinical outcomes.^13^, ^14^

It is well‐established that hematologic and laboratory markers undergo changes following neoadjuvant therapy. Among these markers, PLT and WBC counts are recognized as significant factors in assessing the risk of surgical site infections, wound healing, and anastomotic leakage. Consequently, this underscores their potential role in influencing survival rates, treatment response, and prognosis following colorectal surgery.^5^, ^9^

So far, although several studies already investigated the prognosis role of preoperative hematologic factors, data have been made available specifically on rectal cancer.^13^ On the other hand, these studies have only examined a limited number of hematologic factors. Hence, we conducted the following retrospective study to investigate the association of several blood indices and examine some of the less common factors to explore their relationship with postoperative infectious complications, survival, and recurrence in rectal cancer patients. Moreover, the importance of preoperative hematologic parameters and baseline characteristics in the mentioned postoperative outcomes was evaluated using machine learning algorithms.

## 2 METHODS

### 2.1 Patients and variables

In this retrospective cohort study, all the 323 CRC patients who were referred to Ghaem Hospital in Mashhad (September 23, 2017 to September 22, 2022) who had cancer confirmed in a biopsy of colonoscopy were considered. The demographic and clinical information of the patients was extracted from the registry, and in 2023, all patients were followed up by phone calls. Patients with surgery techniques of exploration (*N* = 7), local excision (*N* = 2), palliative colostomy loop formation (*N* = 12), and perineal colostomy (N = 1) were excluded. In addition, squamous cell carcinoma (*N* = 2), melanoma (*N* = 1), metastatic (*N* = 7), hospital mortality (*N* = 4), and loss to follow‐up (*N* = 10) patients were eliminated. Finally, colon cancer patients (*N* = 77) were excluded. More than 90% of the colon cancer patients did not receive neoadjuvant treatment, whereas neoadjuvant treatment accounted for significant variation in outcomes. All 200 rectal cancer patients have received neoadjuvant chemoradiation therapy, and at least a month after chemoradiation, the surgery was performed. The patients were administered capecitabine 1 g/m^2^ BID as part of their chemotherapy treatment, and underwent a total of 28 sessions of radiation therapy, with a total radiation dose of 5040 rad. All of the study participants had an ASA indexing score of II or III. Before surgery, the physical condition of a patient is assessed using the ASA score. The scale runs from 1 to 6, with higher scores suggesting a higher risk of complications. Until the fifth year following curative surgery, study participants were monitored according to routine rectal cancer surveillance. During the first 2 years after surgery, patients were followed up every 3 months. Subsequently, follow‐up appointments were scheduled every 6 months until the fifth year. Following these times, patients have received yearly phone calls.

Demographic and clinical baseline characteristics of age at diagnosis, gender, nationality (Iranian/non‐Iranian), surgical procedure techniques (abdominoperineal resection/Hartmann's procedure/coloanal anastomosis/ultra‐ilow anterior resection/low anterior resection), ypT and ypN stage (based on TNM staging system), resection margin status, bleeding during surgery, and surgery type (laparotomy/laparoscopy/ conversion) were examined in this study. Preoperative hematologic findings of platelet (PLT) (10^9^/L), platelet distribution width (PDW) (fL), mean platelet volume (MPV) (fL), platelet/lymphocyte ratio (PLR), neutrophil/lymphocyte ratio (NLR), lymphocyte/white blood cell (LWR), platelet/ neutrophil ratio (PNR), and glomerular filtration rate (GFR) (mL/min/1.73^2^) based on MDRD formula, and red cell blood distribution width (RDW‐CV) (fL) were evaluated to determine the association between these factors and the outcomes of mortality, recurrence and infectious surgical complications. These hematologic factors belong to hospital admission time before the surgical procedure—a minimum time interval of 24 h until the surgery has been considered to record these factors. The instruments for analyzing CBC and biochemical parameters were Sysmex KX‐21N and Biotecnica instrument (BT3500), respectively. Quality assurance was conducted in three phases. First, faults in the Levey–Jennings chart were identified. Second, the lab device was checked daily using control samples, and monthly by an external sample control group. Third, the coefficient of variation (CV) of lab results was defined monthly and compared with the licensed range. Additionally, the lab devices were inspected every 6 months by a control checker company named “Advanced Electronic.”

Overall mortality was specified as postoperative mortality (suicide or traumatic mortality did not occur in this study). Recurrence outcomes were defined as locoregional or distant recurrence during follow‐up and infectious surgical complications characterized by surgical site infection, pelvic collection/abscess, or abdominal wall abscess. All data were collected from the colorectal cancer registry (No: 4001728) at Mashhad University of Medical Sciences, Mashhad, Iran.

### 2.2 Statistical analysis

Quantitative normal variables were reported as mean ± standard deviation (SD) and non‐normal variables were described as median (IQR: interquartile range). Categorical characteristics were also expressed in frequency (percentage). After checking relevant assumptions, the chi‐squared test or Fisher's exact test was performed to compare the categorical variables and to compare continuous variables, after checking the normality, the independent two‐sample t‐test or Mann–Whitney test (which one is applicable) was used. The receiver operating characteristics (ROC) curve analysis was utilized to assess the classifying role of hematologic factors in the outcomes of death, recurrence, and surgical infectious complications. The optimal cutoff point of hematologic factors' level was determined through Youden's index. The missing values in our hematologic findings were less than 10%; instead of removing the values, missing values were imputed using the “MICE” package. Demographic and clinical characteristics as only predictors were included in this imputation. In addition, the hematologic findings were considered both as predictors and as variables to be imputed. The SPSS version 26.0 (Chicago, IL, USA) and MedCalc Statistical Software version 22.005 (MedCalc Software Ltd, Ostend, Belgium; https://www.medcalc.org; 2023) were rendered for analysis. The significance level was considered 0.05.

The feature importance analysis was performed by Random Forest (RF), Recursive Feature Elimination, Simulated Annealing, Support Vector Machine (SVM), Decision Tree, and eXtreme Gradient Boosting (XGBoost). K‐fold cross‐validation technique was used in our machine learning approach. After analyzing the feature importance values from each classifier model, we normalized values to illustrate each feature's importance. As well as we averaged the values to calculate the combination feature importance values. Three top models among all models based on accuracy criteria have been selected to demonstrate the most relevant variables for the outcomes of death, recurrence, and surgical infectious complications. If a dataset is unbalanced, the feature impotence analysis tends to overfit the majority outcome. To face this issue in our dataset, the OVUN.SAMPLE function from the “ROSE” package was applied considering a combination of both oversampling and undersampling. R 4.2.3 and RStudio 2023.06.2 and packages including “Random Forest,” “caret,” “e1071,” and “XGBoost” was employed for feature importance analysis.

## 3 RESULTS

The study encompassed 200 eligible patients diagnosed with rectal cancer, exhibiting a mean ± SD age of 54.2 ± 13.8 years. Among these patients, 58% were male. The median (IQR) follow‐up time for all the patients was 22.5 (23) months. The median (IQR) follow‐up value for surviving patients was 23 (24.5) months. Postoperative infectious complications, mortality, and recurrence developed in 30%, 20.5%, and 24% of patients, respectively. Most recurrences in our patients belonged to distant metastasis, which included 70.8% of all recurrences in this cohort. Locoregional and distant recurrence were occurred in 5.5% (*N* = 11) and 17% (*N* = 34) of the studied patients. In addition, 1.5% (*N* = 3) of the patients experienced both kinds of recurrences. The patient's demographic and clinical profiles, stratified based on distinct outcomes of infectious complications, tumor recurrence, and mortality were summarized in Table 1. Notably, the frequency of infectious complications demonstrated a correlation with the surgical procedure administered (*p* < 0.001). Observations from Table 1 elucidated that the incidence of tumor recurrence among patients was significantly influenced by the tumor's depth (denoted as T stage) (*p* = 0.014) and the involvement of the lymph nodes (*p* = 0.001).

**TABLE 1:** The demographic and clinical baseline characteristics of the patients.

Variables	Complicated patients (*N* = 60)	Noncomplicated patients (*N* = 140)	*p*	Recurrent patients (*N* = 48)	Nonrecurrent patients (*N* = 152)	*p*	Dead patients (*N* = 41)	Alive patients (*N* = 159)	*p*
Age at diagnosis, mean ± SD, year	54.3 ± 15	54.2 ± 13.2	0.960	55.7 ± 14.3	53.8 ± 13.7	0.409	63.3 ± 14.3	51.9 ± 12.7	<0.001^a^
Gender, *N* (%)									
Male	34 (56.7)	82 (58.6)	0.803	28 (58.3)	88 (57.9)	0.957	30 (73.2)	86 (54.1)	0.027^a^
Female	26 (43.3)	58 (41.1)		20 (41.7)	64 (42.1)		11 (26.8)	73 (45.9)	
Nationality, *N* (%)									
Iranian	59 (98.3)	135 (96.4)	0.671	48 (100)	146 (96.1)	0.339	39 (95.1)	155 (97.5)	0.605
Non‐Iranian	1 (1.7)	5 (3.6)		0	6 (3.9)		2 (4.9)	4 (2.5)	
Surgery techniques, *N* (%)									
Abdominoperineal resection	31 (51.7)	36 (25.7)	<0.001^a^	19 (39.6)	48 (31.6)	0.322	19 (46.3)	48 (30.2)	0.077
Hartmann's procedure	10 (16.7)	11 (7.9)		4 (8.3)	17 (11.2)		6 (14.6)	15 (9.4)	
Coloanal anastomosis	8 (13.3)	28 (20)		12 (25)	24 (15.8)		8 (19.5)	28 (17.6)	
Ultra‐low anterior resection	10 (16.7)	54 (38.6)		11 (22.9)	53 (34.9)		7 (17.1)	57 (35.8)	
Low anterior resection	1 (1.7)	11 (7.9)		2 (4.2)	10 (6.6)		1 (2.4)	11 (6.9)	
Surgery type, *N* (%)									
Laparotomy	50 (83.3)	112 (80)	0.530	37 (77.1)	125 (82.2)	0.084	34 (82.9)	128 (80.5)	0.363
Laparoscopy	9 (15)	27 (19.3)		9 (18.8)	27 (17.8)		6 (14.6)	30 (18.9)	
Conversion	1 (1.7)	1 (0.7)		2 (4.2)	0		1 (2.4)	1 (0.6)	
T stage, *N* (%)									
T0	15 (25)	36 (25.7)	0.807	6 (12.5)	45 (29.6)	0.014^a^	5 (12.2)	46 (28.9)	0.008^a^
T1	4 (6.7)	5 (3.6)		2 (4.2)	7 (4.6)		0	9 (5.7)	
T2	18 (30)	38 (27.1)		10 (20.8)	46 (30.3)		9 (22)	47 (29.6)	
T3	18 (30)	51 (36.4)		24 (50)	45 (29.6)		22 (53.7)	47 (29.6)	
T4	5 (8.3)	10 (7.1)		6 (12.5)	9 (5.9)		5 (12.2)	10 (6.3)	
N stage, *N* (%)									
N0	41 (68.3)	95 (67.9)	0.862	23 (47.9)	113 (74.3)	0.001^a^	20 (48.8)	115 (72.3)	0.013^a^
N1	8 (13.3)	24 (17.1)		9 (18.8)	23 (15.1)		8 (19.5)	24 (15.1)	
N2	6 (10)	11 (7.9)		10 (20.8)	7 (4.6)		7 (17.1)	10 (6.3)	
NX	5 (8.3)	10 (7.1)		6 (12.5)	9 (5.9)		6 (14.6)	10 (6.3)	
Resection margin status, *N* (%)									
R0	52 (86.7)	127 (90.7)	0.392	43 (89.6)	136 (89.5)	0.983	32 (78)	147 (92.5)	0.018^a^
R1	8 (13.3)	13 (9.3)		5 (10.4)	16 (10.5)		9 (22)	12 (7.5)	
Bleeding during surgery, *N* (%)									
None	55 (91.7)	134 (95.7)	0.311	44 (91.7)	145 (95.4)	0.300	38 (92.7)	151 (95)	0.699
Yes	5 (8.3)	6 (4.3)		4 (8.3)	7 (4.6)		3 (7.3)	8 (5)	

^a^ Significant at *α* = 0.05 NX: lymph node was not identified R1: microscopic involved resection margin.

Furthermore, regarding the mortality outcome, alongside tumor depth (*p* = 0.008) and lymph node involvement (*p* = 0.013), the status of resection margin involvement emerged as a significant determinant of patient mortality (*p* = 0.018). Strikingly, deceased patients exhibited an average age markedly higher than their living counterparts (*p* < 0.001). This finding is paralleled by the observation that a substantial 73.2% of the deceased patients were male (*p* = 0.027).

**FIGURE 1:**
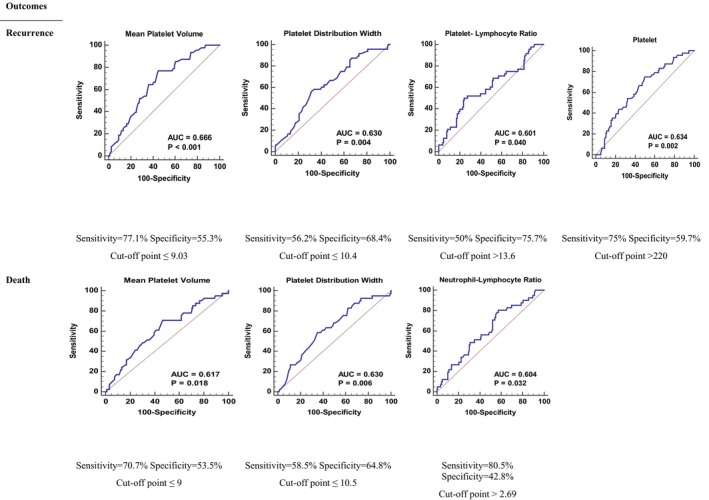
ROC curve analysis results with reported cutoff points, sensitivity, and specificity.

Pivotal factors that significantly contribute to patient classification to specified outcomes were illustrated in Figure 1, accompanied by their respective cutoff values, sensitivity, and specificity levels.

A NLR exceeding 2.69 (*p* = 0.032), MPV less than or equal to 9 fL (*p* = 0.018), and PDW less than or equal to 10.5 fL (*p* = 0.006) is associated with mortality. Likewise, certain parameters were associated with tumor recurrence. Platelet counts greater than 220 10^9^/L (*p* = 0.002), PDW less than or equal to 10.4 fL (*p* = 0.004), MPV less than or equal to 9 fL (*p* < 0.001), and PLR exceeding 13.6 (*p* = 0.040) was indicative of an increased likelihood of recurrence in patients. All factors examined in this study were not significant classifiers for the outcome of surgical infectious complications.

The patient cohort was divided into two groups based on the established cutoff values of significant hematological factors. Subsequently, the study assessed the homogeneity of the frequency distribution of these dichotomous factors between significant variables revealed in Table 1.

Among hematologic factors associated with cancer recurrence, only the categorized MPV variable was associated with nodal involvement (*p* = 0.016). About 82% of N2 patients and 73.3% of patients with unspecified lymph nodes had MPV with equal or lower values of 9.03 fL. Other hematological variables associated with cancer recurrence were independent of the T‐stage and N‐stage variable levels (All *p*‐values > 0.05).

The mean age of patients was not significantly different between dichotomous factors of MPV (*p* = 0.507), NLR (*p* = 0.318), and PDW (*p* = 0.513). The frequency distribution of gender, T stage, N stage, and resection margin status of patients was homogeneous between NLR and PDW levels (All *p*‐values > 0.05). Whereas, MPV was associated with lymph node involvement (*p* = 0.035). More than 70% of N2 and NX patients had MPV equal to or less than 9 fL. Additionally, MPV variables were independent of gender (*p* = 0.432), T stage (*p* = 0.059), and resection margin status (*p* = 0.142).

**FIGURE 2:**
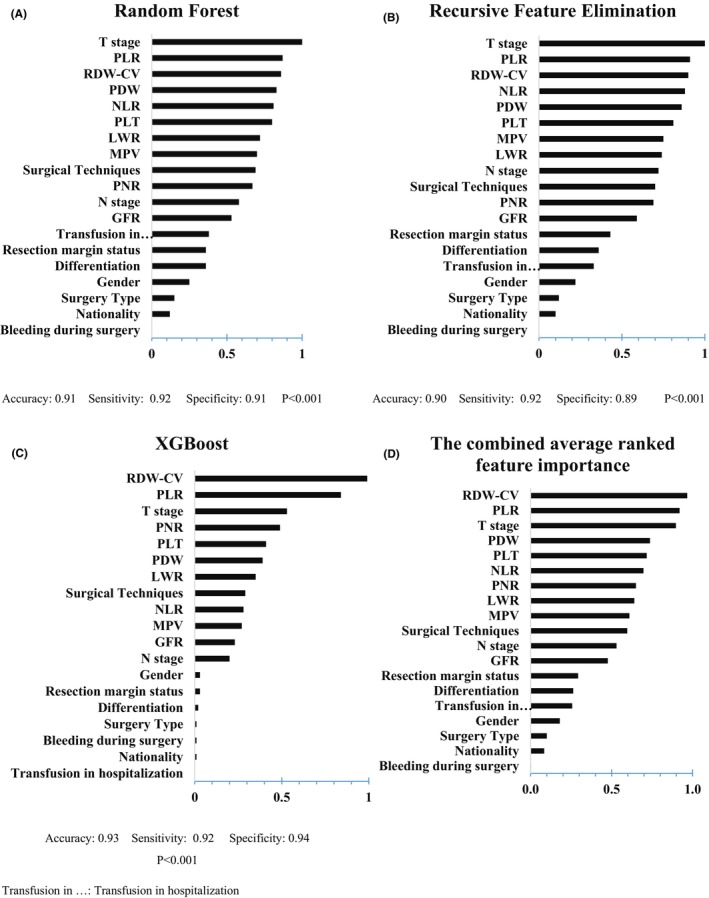
Results of normalized feature importance analysis from Random Forest (A), Recursive Feature Elimination (B), and XGBoost (C), and the combined average ranked feature importance (D) in death outcome.

**FIGURE 3:**
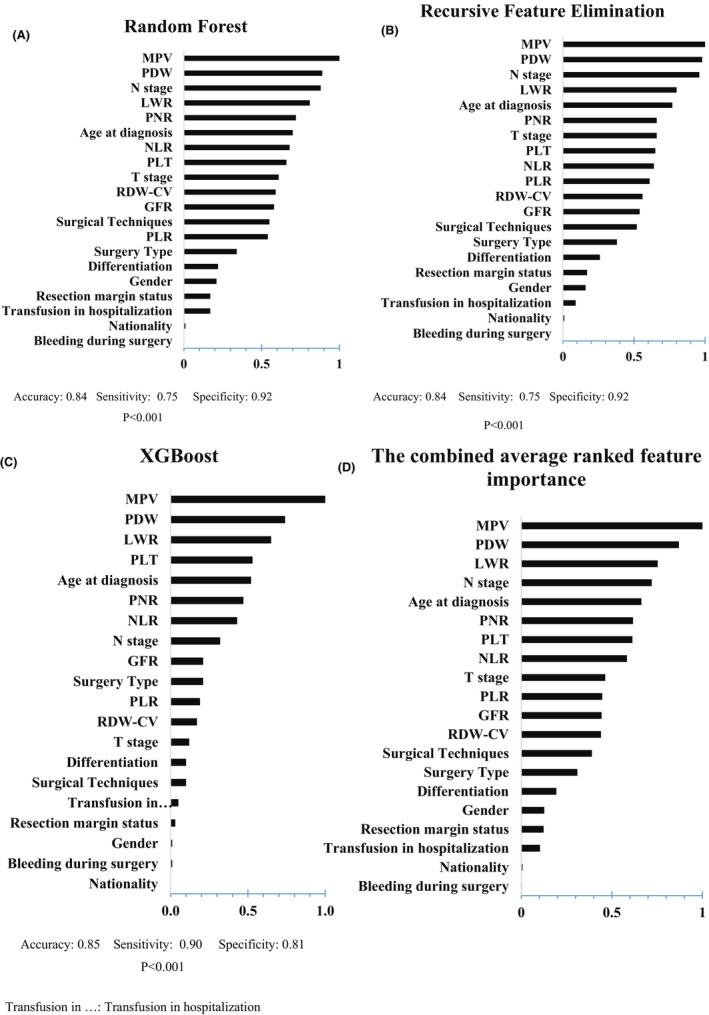
Results of normalized feature importance analysis from Random Forest (A), Recursive Feature Elimination (B), and XGBoost (C), and the combined average ranked feature importance (D) in recurrence outcome.

**FIGURE 4:**
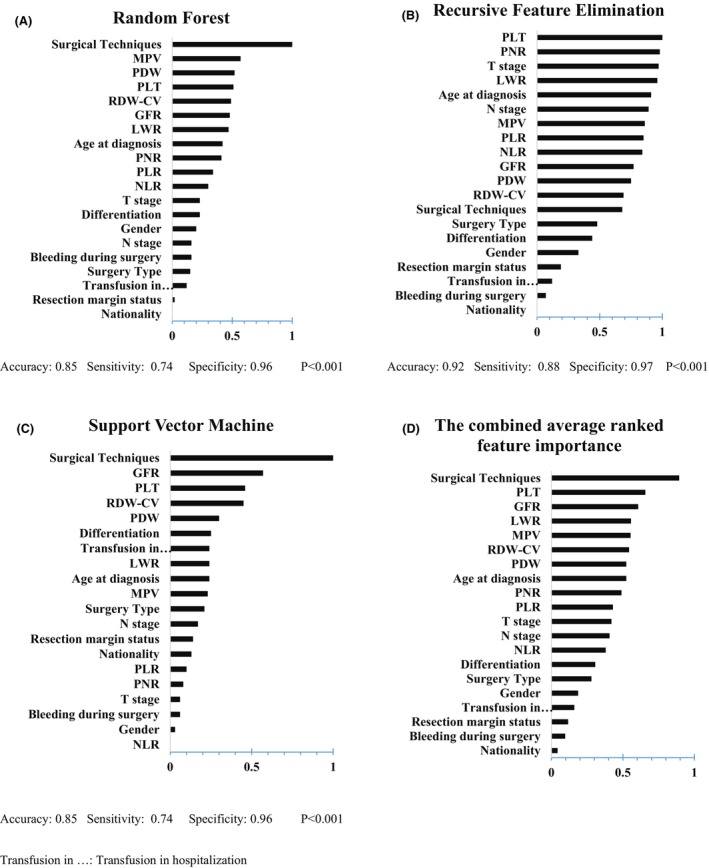
Results of normalized feature importance analysis from Random Forest (A), Recursive Feature Elimination (B), and SVM (C), and the combined average ranked feature importance (D) in surgical infectious complication outcome.

Figures 2, 3, 4 show the results of the ranked feature importance from three top ML models based on high accuracy, and their combination. In the outcome of death (Figure 2), the MPV, PDW, and NLR variables, known as significant classifiers in ROC analysis, were also among the top 10 important features in the combination of ML models. The presence of RDW‐CV was considerable in the most important features. In ROC analysis, the *p*‐value of this variable was borderline (*p* = 0.052). It is not surprising that the T stage was among the most important features. It was also related to death in Table 1.

For the outcome of recurrence (Figure 3), the combination results of ML models were in line with ROC analysis. MPV, PDW, PLR, and PLT were part of the top 10 important features. Along with other important hematological variables, age at diagnosis and involvement of lymph nodes were the most important features in recurrence prediction.

In the outcome of infectious complications after surgery (Figure 4), surgical technique was recognized as the most important feature based on ML models. This finding confirmed Table 1's results. The presence of GFR in the most important features of postoperative infectious complications is a notable point.

## 4 DISCUSSION

We performed a retrospective study to determine the association of preoperative hematologic parameters with surgical outcomes following rectal cancer resection. In recent years, ML algorithms have been also used to predict the accurate prognosis of CRC. The adoption of data‐intensive ML methods may lead to more evidence‐based decision‐making in surgery.^15^, ^16^

Our results demonstrated that the infectious complications following rectal cancer surgery were significantly influenced by the surgery technique. However, none of the studied hematologic parameters had a significant association with the occurrence of these complications. The results of the ML were in line with ROC analysis and the surgical technique was the most important factor in the incidence of infectious outcomes. At the same time, machine learning showed that in addition to the surgical technique, some hematological parameters such as PLT and GFR may also be among the three top important factors. However, there is no evidence in the literature about the association of these factors with an increased risk of infectious complications.^17^, ^18^, ^19^

In this study, ROC analysis demonstrated that in addition to the baseline characteristics of age, gender, T stage, N stage, and resection margin status, multiple hematologic parameters including MPV, NLR, and PDW were predictors of mortality after rectal cancer surgery.

Regarding the relationship between the MPV and the overall survival (OS) of CRC patients, various studies have been conducted, which have resulted in contradictory results. However, such studies on rectal cancer are very limited. In a study by Chang et al. on metastatic CRC patients treated with first‐line chemotherapy, it was reported that preoperative MPV <9.75 fL was associated with poorer OS.^20^ Qian et al. also reported that CRC patients with a post‐/preoperation MPV ratio <1 had poorer OS.^21^ On the contrary, some other reports in rectal cancer have shown that high MPV level is associated with shorter OS. To illustrate, Jonnada et al. verified the optimal cutoff value of 9.35 fL for MPV and revealed that rectal cancer patients with MPV <9.35 fL had a significantly better 5‐year OS than patients with MPV >9.35 fL.^22^ Demir et al. also observed that rectal cancer patients with MPV ≥7.65 fL had a worse prognosis than patients with a low level of MPV <7.65 fL.^23^ The ideal cutoff value in the present study was 9.03 in ROC analysis, and unlike the mentioned rectal cancer studies, the MPV ≤9.03 was associated with higher mortality.

The prognostic role of NLR has been investigated in gastrointestinal solid cancers,^24^, ^25^, ^26^, ^27^ but few and conflicting findings have been reported in rectal cancer.^13^, ^28^, ^29^ Carruthers et al. demonstrated that NLR ≥5 was significantly associated with longer OS, and the median survival in patients with elevated NLR was nearly threefold.^29^ On the other hand, in a meta‐analysis by Dong et al. which reported the findings of seven studies, it has been illustrated that a higher NLR was associated with poor OS in rectal cancer patients. Moreover, in some studies, the results were strikingly different as it had been shown that the NLR did not correlate with survival outcomes in rectal cancer cases.^13^, ^30^ Our results about the NLR were in line with the mentioned meta‐analysis study,^28^ and we found that NLR >2.69 was associated with poorer OS.

Several studies have reported that PDW is significantly associated with longer OS in various malignancies.^31^, ^32^, ^33^ Nevertheless, there are few and conflicting studies which have investigated the predictive value of preoperative PDW in CRC and particularly rectal cancer patients. Recently, Benek et al. demonstrated that elevated PDW was associated with poorer OS in CRC patients.^34^ Similarly, Song et al. showed that increasing PDW was associated with worse OS in non‐metastatic CRC patients.^35^ On the other hand, Qian et al. revealed that preoperative PDW does not correlate with patient outcomes.^21^ Wang et al. also reported no significant association between PDW and OS in rectal cancer patients.^36^ Unlike the previous studies, we found that PDW ≤10.5 fL was associated with worse OS. Although our result on PDW was not in line with the mentioned studies, some literature has shown similar results to ours. To illustrate, Sakin et al. showed that decreased PDW appears to be an unfavorable prognostic factor in early colon cancer, particularly in patients with Stage III disease.^37^

Although the MPV, PDW, and NLR were defined as classifiers of death in the ROC analysis, ML results considered RDW‐CV as the most important hematologic factor in the outcome of death. It should be considered that in ROC analysis, the *p*‐value of RDW‐CV was borderline (*p* = 0.052). RDW‐CV has been also studied as a potential prognostic marker in various cancer such as lung, liver, esophagogastric, breast, and particularly CRC.^9^, ^38^ In addition, it is not surprising that the T stage was among the three top important features in ML results. On the other hand, regarding the crucial role of platelets in tumor growth and tumor invasion,^39^ the presence of PLT and the other platelet‐associated indicators among the most important factors was not far from expected. Therefore, the results of the ROC analysis and ML on death outcomes do not seem contradictory.

Increased PLT leads to cancer progression and metastasis by shielding circulating tumor cells from immune surveillance and killing.^40^ Toiyama et al. reported that PLT was the only biomarker independently predicting disease‐free survival (DFS) and recurrence in locally advanced rectal cancer patients who underwent neoadjuvant chemoradiation treatment followed by surgery.^41^ Cravioto‐Villanueva et al. also demonstrated that preoperative PLT >350,000/dL was associated with the development of distant metastasis in patients with rectal cancer.^40^ In the present study, it was shown that PLT >220,000/dL was associated with the recurrence of rectal cancer. Moreover, the other platelet‐associated indicators including MPV, PDW, and PLR could be considered as classifiers of the recurrence. Scholars have illustrated that alteration in the mentioned indices is associated with the recurrence of CRC.^35^, ^37^ Nevertheless, the literature focusing on the predictive effects of the mentioned platelet‐related parameters in rectal cancer is scarce and somewhat contradictory to our results. To illustrate, Jonnada et al. reported longer DFS in Stage III rectal cancer patients with MPV <9.35 fL.^22^ Demir et al. also revealed that rectal cancer patients with MPV <7.65 had a longer DFS.^23^ On the other hand, in a recent study by Wang et al. MPV and PDW did not show any statistical association with DFS in locally advanced rectal cancer patients.^36^ At the same time, we found that MPV≤9.03 fL and PDW≤10.4 fL were associated with the recurrence in rectal cancer cases. Regarding the PLR, Portale et al. showed no association between the PLR and DFS after rectal cancer surgery,^13^ while Sung Woo et al., showed that high PLR (>92.88) was an indicator of a favorable RFS outcome in locally advanced rectal cancer.^42^ However, in the present study, PLR was one of the classifiers of recurrence, and PLR >13.6 was indicative of an increased likelihood of recurrence. The ML results were also partially aligned with the ROC analysis results so that the MPV and PDW were among the three top important factors in recurrence outcome. Moreover, PLT and PLR were among the 10 most important parameters associated with the recurrence.

Our results revealed that T and N stages were significantly associated with both recurrence and OS, which is consistent with previous studies.^36^, ^43^ Furthermore, OS was also significantly associated with age, gender, and resection margin status. As mentioned before, surgery technique was the only prognostic factor that had a significant association with surgical infectious complications. Moreover, examining the relationship between the obtained classifiers of recurrence and death, demonstrated that in both outcomes, only categorized MPV was associated with nodal involvement, and there was no association between the other hematologic parameters and the studied baseline characteristics.

In this study, although the results of ROC analysis and ML were aligned, minor differences were also evident in their results. These differences are probably due to the difference in the nature of the analyses as well as the difference in the accuracy of the models. ROC curves were calculated for each hematological parameter and each outcome separately, while feature importance analysis measured the importance of all pathological, demographic, and hematological factors together. Also, several studies have been conducted on the importance of hematological factors in predicting clinical outcomes in CRC patients using regression methods. However, in the present study, several parameters have been considered. So, due to the multicollinearity issue, it was not possible to use regression methods, and therefore classification models were considered.

This study has several limitations. The main limitation of our study relates to its retrospective nature and the limited sample size. Although our cutoff values were obtained using valid statistical analyses, these values need to be validated in studies of larger scales. Moreover, hematological parameters might be influenced by drugs, such as aspirin, or nontumorous diseases such as coronary artery disease, or metabolic syndrome. However, our study has many strengths including long‐term follow‐ups after surgery, a homogenous patient population, and the examination of a large number of well‐studied prognostic factors accompanied by less common factors using multiple comprehensive analyses. In various studies that have been conducted on preoperative hematologic factors and their association with rectal cancer prognosis, different and sometimes contradictory results are observed. The differences between the enrolled patients and the stage of their rectal cancer might be the reason for this controversy. In addition, when measuring hematologic factors, there are several confounding factors that can influence the results. In our study, we have taken measures to control these factors, such as considering the time from neoadjuvant therapy and using the same device to assess the complete blood count for all patients. Therefore, we suggest that future studies should also consider these factors to enable better comparison of results. Multicenter, prospective studies with larger sample sizes could also be designed in the future to overcome this controversy.

## 5 CONCLUSION

In our study, in addition to the several baseline characteristics, increased NLR, and decreased MPV and PDW were found to be the classifiers of mortality. RDW‐CV along with the other platelet‐related indices were also the most important hematologic factors associated with overall survival. Recurrence had an association with T stage, N stage, increased PLT and PLR, and decreased MPV and PDW, while none of the studied hematologic parameters showed an association with the outcome of surgical infectious complications. However, the surgery technique was significant and the most important factor that affected this outcome. We believe that these preoperative hematologic biomarkers despite being inexpensive and accessible, can provide important data for studying the response to surgical treatment in rectal cancer patients. Therefore, it is recommended that clinicians consider the mentioned parameters for identifying and screening high‐risk patients. However, further research is warranted to explore this matter in greater depth in future studies.

## AUTHOR CONTRIBUTIONS

**Ala Orafaie:** Investigation (supporting); writing – original draft (lead). **Fatemeh Shahabi:** Methodology (lead). **Ali Mehri:** Writing – review and editing (supporting). **Majid Ansari:** Writing – review and editing (equal). **Sajjad Kasraeifar:** Investigation (equal). **Mahdie Ghiyasi:** Investigation (equal). **Maryam Saberi‐Karimian:** Investigation (supporting). **Abbas Abdollahi:** Supervision (lead). **Seyyed Mohammad Tabatabaei:** Methodology (equal); supervision (equal).

## FUNDING INFORMATION

This research did not receive any specific grant from funding agencies in the public, commercial, or not‐for‐profit sectors.

## CONFLICT OF INTEREST STATEMENT

The authors declare no conflict of interest.

## ETHICS STATEMENT

The study was approved by the Mashhad University of Medical Sciences Ethical Committee (IR.MUMS.IRH.REC.1401.030) and was performed in accordance with the Declaration of Helsinki. Mashhad University of Medical Sciences Ethical Committee has waived the informed consent due to the retrospective nature of the study.

## CONSENT FOR PUBLICATION

Not applicable.

## Data Availability

The datasets used and/or analyzed during the current study are available from the corresponding author upon reasonable request.
